# Effectiveness and tolerability of transdermal buprenorphine patches: a multicenter, prospective, open-label study in Asian patients with moderate to severe chronic musculoskeletal pain

**DOI:** 10.1186/s12891-017-1664-4

**Published:** 2017-08-04

**Authors:** Do Heum Yoon, Seong-Il Bin, Simon Kin-Cheong Chan, Chun Kee Chung, Yong In, Hyoungmin Kim, Juan Javier Lichauco, Chi Chiu Mok, Young-Wan Moon, Tony Kwun-Tung Ng, Ester Gonzales Penserga, Dong Ah Shin, Dora You, Hanlim Moon

**Affiliations:** 1 Department of Neurosurgery, Spine and Spinal Cord Institute, Yonsei University College of Medicine, Severance Hospital, 134 Shinchon-dong Seodaemun-gu, Seoul, 120–752 South Korea; 2 Department of Orthopedic Surgery, Asan Medical Center, Seoul, South Korea; 3 Pain Management Unit, Department of Anaesthesiology and Intensive Care, Prince of Wales Hospital, The Chinese University of Hong Kong, Hong Kong, SAR China; 4 Department of Neurosurgery, Seoul National University Hospital, Seoul National University College of Medicine, Seoul, South Korea; 5 Department of Brain and Cognitive Science, Seoul National University College of Natural Science, Seoul, South Korea; 6 Department of Orthopaedic Surgery, Seoul St. Mary’s Hospital, College of Medicine, The Catholic University of Korea, Seoul, South Korea; 7 Department of Orthopedic Surgery, Seoul National University Hospital, Seoul National University College of Medicine, Seoul, South Korea; 8 Section of Rheumatology, Department of Medicine, St. Luke’s Medical Center, Manila, Philippines; 9 Department of Medicine, Tuen Mun Hospital, Hong Kong, SAR China; 10 Department of Orthopedic Surgery, Samsung Medical Center, Sungkyunkwan University School of Medicine, Seoul, South Korea; 11 Pain Management Unit, Department of Anaesthesia and Intensive Care, Tuen Mun Hospital, Hong Kong, SAR China; 12 Section of Rheumatology, Department of Medicine, University of the Philippines College of Medicine-Philippine General Hospital, Manila, Philippines; 13 Mundipharma Pte Ltd, Asia Square Tower 2, Singapore

**Keywords:** Transdermal buprenorphine, Asian, Chronic non-malignant pain, Musculoskeletal, Pain score, Quality of life, Sleep quality, Effectiveness, Tolerability

## Abstract

**Background:**

We examined the effectiveness and tolerability of transdermal buprenorphine (TDB) treatment in real-world setting in Asian patients with musculoskeletal pain.

**Methods:**

This was an open-label study conducted in Hong Kong, Korea, and the Philippines between June 2013 and April 2015. Eligible patients fulfilled the following criteria: 18 to 80 years of age; clinical diagnosis of osteoarthritis, rheumatoid arthritis, low back pain, or joint/muscle pain; chronic non-malignant pain of moderate to severe intensity (Box-Scale-11 [BS-11] pain score ≥ 4), not adequately controlled with non-opioid analgesics and requiring an opioid for adequate analgesia; and no prior history of opioid treatment. Patients started with a 5 μg/h buprenorphine patch and were titrated as necessary to a maximum of 40 μg/h over a 6-week period to achieve optimal pain control. Patients continued treatment with the titrated dose for 11 weeks. The primary efficacy endpoint was the change in BS-11 pain scores. Other endpoints included patients’ sleep quality and quality of life as assessed by the 8-item Global Sleep Quality Assessment Scale (GSQA) questionnaire and the EuroQol Group 5-Dimension Self-Report Questionnaire-3 Level version (EQ-5D-3 L), respectively. Tolerability was assessed by collecting adverse events.

**Results:**

A total of 114 eligible patients were included in the analysis. The mean BS-11 score at baseline was 6.2 (SD 1.6). Following initiation of TDB, there was a statistically significant improvement in BS-11 score from baseline to visit 3 (least squares [LS] mean change: -2.27 [95% CI -2.66 to −1.87]), which was maintained till the end of the study (visit 7) (LS mean change: −2.64 [95% -3.05 to −2.23]) (*p* < 0.0001 for both). The proportion of patients who rated sleep quality as ‘good’ increased from 14.0% at baseline to 26.9% at visit 6. By visit 6, the mean EQ VAS score increased by 7.7 units (SD 17.9). There were also significant improvements in patients’ levels of functioning for all EQ-5D-3 L dimensions from baseline at visit 6 (*p* < 0.05 for all). Seventy-eight percent of patients reported TEAEs and 22.8% of patients discontinued due to TEAEs. TEAEs were generally mild to moderate in intensity (96.5%).

**Conclusions:**

TDB provides effective pain relief with an acceptable tolerability profile over the 11-week treatment period in Asian patients with chronic musculoskeletal pain. More studies are needed to examine the long-term efficacy and safety of TBD treatment in this patient population.

**Trial registration:**

ClinicalTrials.gov NCT01961271. Registered 7 October 2013 (retrospectively registered; first patient was enrolled on 28 June 2013 and last patient last visit date was 26 Apr 2015).

## Background

Musculoskeletal disorders are among the most common causes of chronic non-malignant pain in adults. Unrelieved pain has significant physiological and psychological impact on patients, affecting their daily quality of life as well as increasing their financial burden [1]. A comprehensive approach encompassing pharmacological and physical therapies, tailored to the individual needs of patients, is needed to reduce pain, promote functional recovery, and improve overall quality of life [2].

Systematic reviews examining evidence on the use of opioid analgesics for the treatment of chronic non-malignant pain suggested these are efficacious in treating pain of various etiologies [3, 4]. However, opioid treatment is associated with typical opioid-induced side effects and potential risk of opioid abuse which can lead to addiction, severe respiratory depression, or even death [3–5]. Guidelines recommend the use of opioid analgesics for the treatment chronic non-malignant pain in patients whose pain persists despite optimized non-opioid treatment. They stress the importance of careful selection of patients who are not at risk of opioid abuse or diversion and ongoing monitoring, together with balancing the goal of achieving pain relief with the risks for opioid abuse or addiction when prescribing treatment [6–8]. Opioid formulations with extended-release (ER) and tamper-resistant properties offer the advantage of achieving analgesia while minimizing risks for opioid abuse or addiction [9–11]. Unlike immediate-release (IR) formulations, ER formulations provide relatively consistent and prolonged plasma drug levels with fewer peak-to-trough fluctuations and lower peak plasma concentration. This results in prolonged analgesia with less frequent dosing and reduced risks of opioid overdose, respiratory depression, and opioid addiction. In addition, the longer time to peak plasma concentration and tamper-resistant features make the formulations less desirable for abuse compared with IR formulations [9–11].

Transdermal buprenorphine (TDB) patches (marketed under different names depending on the country: Norspan®, Sovenor®, Butrans®, or Restiva®), in three dose strengths: 5 μg/h, 10 μg/h, or 20 μg/h, have been developed [12]. It is licensed for the treatment of moderate to severe chronic pain that do not respond to non-opioid analgesics [13–15]. Its active ingredient, buprenorphine, is a potent opioid analgesic that acts primarily as a partial agonist at the μ-opioid receptor [16]. TDB offers several advantages over typical full μ-opioid receptor agonists in treating chronic pain. First, its unique partial agonist activity induces a ceiling effect for respiratory depression but not for analgesia, resulting in a reduced risk for this potentially fatal adverse event compared to other full opioid agonists [17, 18]. Next, TDB has a lower propensity for opioid abuse or addiction than typical full opioid agonists [9, 17] and its transdermal matrix makes it difficult to extract the substance for non-medical use [10]. In addition, unlike most full opioid agonists which are eliminated primarily in urine, buprenorphine is mainly excreted through the feces and does not accumulate in the body. This makes it more suitable and convenient to use than full opioid agonists because it does not require special dose adjustments in patients with compromised renal function, such as the elderly or renal patients [18–21]. Next, TDB releases a steady and continuous dose of buprenorphine over a period of up to seven days which confers the convenience of once-weekly dosing. Given that prescribing of analgesics in patients with musculoskeletal disorders may be complicated by comorbid conditions and polypharmacy, the extended analgesia duration offered by TDB allows for less frequent dosing and reduces pill burden compared with other oral opioid agonists. This may help improve patient acceptability and adherence. Besides these, TDB can be used by patients who have difficulty swallowing, or have gastrointestinal disorders, or preexisting nausea and vomiting and are unable to take oral opioid analgesics [22].

TDB has demonstrated good efficacy and an acceptable tolerability profile in patients with chronic non-malignant pain in randomized controlled trials [23–25]. Pain intensity and sleep disturbance were considerably reduced and patients experienced improved physical function and quality of life after treatment. TDB was tolerated by the majority of the patients in these studies [23–25]. TDB was noted to be non-inferior to other opioid analgesics in reducing pain. Apart from application site reactions that were typical of transdermal delivery systems, TDB has an AE profile that is comparable with the other opioid analgesics [23, 25]. However, real-world data on treatment of chronic pain with TDB is limited [26]. To date, there are no multinational studies examining the use of TDB in Asian patients with chronic non-malignant pain. This study aimed to assess the effectiveness and tolerability of TDB in real-life clinical settings in Asian patients who were suffering from moderate to severe musculoskeletal pain.

## Methods

### Study design

**Fig. 1:**
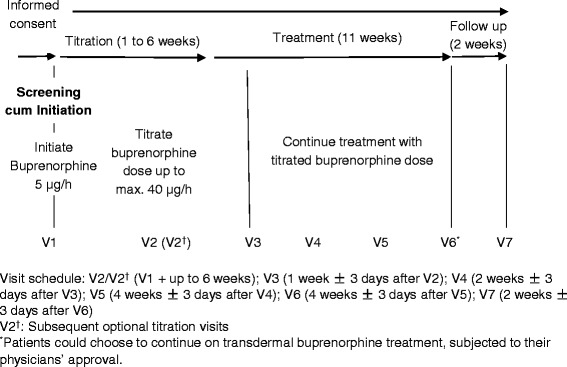
Study design

This was a prospective, multicenter, open-label, single-arm study conducted in 16 hospital sites across three countries or territories in Asia (Hong Kong, Korea, and the Philippines) between June 2013 and April 2015. The study comprised a screening and baseline visit (visit 1) to assess patient eligibility and collect baseline data, followed by a titration period of up to six weeks, during which patients received the study medication and their dose was adjusted as necessary to achieve optimal pain control. Patients then entered an 11-week treatment period and attended a follow-up visit two weeks after completion of treatment. The study consisted of at least seven scheduled visits at specified intervals (Fig. 1).

### Patients

Inclusion criteria were as follows: (i) 18–80 years of age; (ii) clinical diagnosis of osteoarthritis, rheumatoid arthritis, low back pain, or joint/muscle pain; and (iii) chronic moderate to severe pain [defined as a score of ≥4 on the Box Scale-11 (BS-11) pain scale], not adequately controlled with non-opioid analgesics and requiring an opioid for adequate analgesia. Exclusion criteria included the following: (i) pregnant or lactating females; (ii) females of childbearing potential who were not willing to use appropriate contraception during the study; (iii) current diagnosis or history of cancer (except basal cell carcinoma); (iv) previous surgery or required surgery; (v) history of alcohol or drug abuse, or behaviors suggestive of addiction or substance abuse; (vi) presence of any contraindication to the study medication; (vii) other chronic conditions that required frequent analgesic therapy; (viii) history of allergy to analgesic agents; (ix) history of prior treatment with study medication; (x) history of opioid treatment; (xi) current or history of steroid treatment; and (xii) deemed unsuitable for participation by the study physician.

### Study treatment

7-day TDB patches, available in three dose strengths: 5 μg/h, 10 μg/h, and 20 μg/h, were used in this study. Patients started with a 5 μg/h buprenorphine patch at the baseline visit and were titrated as necessary to a maximum of 40 μg/h over a 6-week period to achieve optimal pain relief. Dose titration and patch application were carried out according to the instructions stated in the local summary of product characteristics or patient information leaflet [13–15]. Patients were prescribed standard recommended doses of rescue analgesics as needed. Antiemetics and laxatives were also permitted, if required. The optimal buprenorphine dose was determined at the physician’s discretion based on patient’s need for supplemental pain relief and analgesic response to the patch. Patients wore the patch continuously for 7 days before titration to the next dose was considered; however, the dose could be increased earlier, after three days of application if patients did not experience adequate analgesia despite administration of rescue analgesics. Buprenorphine dose was increased gradually in increments of 5 μg/h either with a patch of higher dose strength or with a combination of patches. No more than two patches were applied at the same time and a new skin site was selected for application. Patients who achieved optimal pain control entered the treatment period, which lasted for 11 weeks. During this period, the physicians adjusted patients’ doses as necessary to maintain optimal pain control. Patients stopped TDB treatment when they completed the 11-week treatment. However, they could choose to continue on TDB treatment, subjected to their physicians’ approval.

### Study assessments

Patients’ demographics and disease characteristics were collected at visit 1. Information relating to concomitant illness was collected throughout the study. Concomitant illness was defined as any medical condition, other than the primary condition, that occurred within the last five years prior to study entry or during the course of the study.

#### Primary efficacy assessment

The primary efficacy assessment was the BS-11 pain score. Before the start of treatment (visit 1) and at each subsequent visit, patients rated their pain level on the BS-11 scale, ranging from a scale of 0 (no pain) to 10 (unbearable pain) [27].

#### Secondary efficacy assessments

The secondary efficacy assessments were as follows:

1. Global Sleep Quality Assessment Scale (GSQA)
  Before treatment (visit1) and at the end of treatment (visit 6) and the follow-up visit (visit 7), patients evaluated their overall sleep quality, degree of sleep disturbance, and sleep duration using the 8-item GSQA scale questionnaire. The GSQA scale ranges from 0 to 10, with higher scores indicating a higher degree of sleep disturbance.
2. EuroQol Group 5-Dimension Self-Report Questionnaire-3 Level Version Survey (EQ-5D-3 L questionnaire)
  Patients assessed their quality of life using the EQ-5D-3 L questionnaire at visits 1, 6, and 7. The questionnaire includes the EQ-5D visual analogue scale (EQ VAS) where patients rated their overall health state on a scale ranging from 0 (worst imaginable health state) to 100 (best imaginable health state). It also includes the descriptive system comprising the following five dimensions: mobility, self-care, usual activities, pain or discomfort, and anxiety or depression where patients indicated their levels of functioning at one of the three levels: no problems, some problems, and extreme problems [28].
3. Global Impression of Change Assessment
  Both patients and physicians evaluated the change in overall pain condition since the initiation of treatment. They completed the Patient Global Impression of Change questionnaire [29] and the Physician Global Impression of Change questionnaire [30], respectively at visit 6. The change was rated on a 7-point scale. A score of 1 indicates “very much improved”, 2 “much improved”, 3 “minimally improved”, 4 “no change”, 5 “minimally worse”, 6 “much worse”, and 7 “very much worse”.
4. Concomitant rescue medication use
  Patients recorded their rescue medication intake in the patient diary throughout the study.

Local language version of all questionnaires was used in each country or territory.

#### Safety assessments

The physicians recorded all adverse events (AEs) that occurred during the study. Vital signs measurements and physical examination were performed at visits, 1, 6, and 7.

### Statistical analysis

#### Sample size estimation

The sample size was estimated based on the results of previous studies [23–25]. Assuming a dropout rate of 20%, it was calculated that at least 53 patients had to be recruited from each country to detect differences in BS-11 scores from baseline for each country at 90% statistical power and at the 5% significance level. As recruiting was progressing more slowly than expected, it was decided to evaluate the overall change in pain scores for the combined study population to ensure sufficient power. Therefore, a total of 119 patients were recruited from the participating countries.

#### Efficacy analysis

The primary efficacy endpoint was the change in BS-11 pain scores between baseline and each visit. A linear mixed model-repeated measures analysis was performed to estimate the least squares (LS) mean change in scores from baseline and 95% confidence intervals (CIs). Baseline score, visit, and country or territory were included as covariates in the model.

Secondary endpoints evaluated included: (i) GSQA overall sleep quality rating, sleep disturbance scores, and sleep duration; (ii) EQ VAS score and ratings for individual EQ-5D-3 L dimensions; (iii) patients’ and physicians’ Global Impression of Change scores; and (iv) prescribed concomitant rescue medications. The Bowker-McNemar test was used to compare patients’ ratings for their overall sleep quality and individual ED-5D-3 L dimensions between visits. Parametric tests were performed where assumptions of normality were met. Otherwise, nonparametric tests were used. The Wilcoxon signed-rank test was used to compare patients’ GSQA sleep disturbance scores and sleep duration between visits, and to compare patients’ and physicians’ Global Impression of Change scores. The paired t-test was used to compare EQ VAS scores between visits. Rescue medication use was summarized using descriptive statistics.

The intent-to-treat (ITT) population was the primary population for efficacy analysis. The efficacy data were analyzed using the per-protocol (PP) population to provide confirmation. The ITT population included eligible patients who received at least one dose of study medication and had pre- and at least one post-intervention assessment of efficacy variables. The PP population was defined as a subset of the ITT population who had completed all the visits without major protocol deviation or violation. All analyses were conducted using available data; no imputation was performed for missing data. A *p*-value of less than 0.05 was considered as statistically significant.

#### Safety analysis

Key safety endpoints evaluated included the incidence of treatment-emergent adverse events (TEAEs) and incidence of TEAEs leading to discontinuation, as well as changes in vital signs and physical examination parameters from baseline to visits 6 and 7. A TEAE was defined as any AE with an onset date on or after the first dose of TDB. Medications prescribed for prevention or for treatment of AEs associated with the study medication were also evaluated. Safety results were descriptively summarized for the safety population. The safety population included eligible patients who received at least one dose of study medication and had at least one safety follow up. All statistical analyses were performed using SAS software, version 9.3 (SAS Institute, Inc., USA).

## Results

### Patient demographics and baseline characteristics

**Fig. 2:**
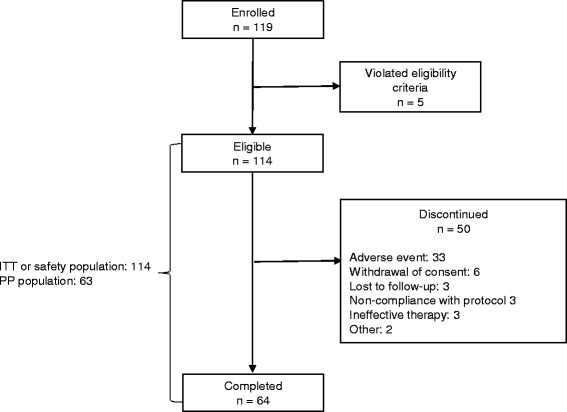
Flow of patients through the study

The flow of patients through the study is shown in Fig. 2. One hundred and nineteen patients were enrolled into the study, five of whom did not meet the eligibility criteria and were excluded from the analysis. Among the remaining one hundred and fourteen eligible patients who received TDB treatment, 64 patients (56.1%) completed the study and 50 (43.9%) discontinued from the study. The reasons for discontinuation are summarized in Fig. 2. All 114 patients were included in the ITT population and safety population. The PP population included 63 patients who completed the study and had no major protocol deviation or violation.

Patients’ demographics and characteristics at study entry are summarized in Table 1. Fifty-four percent of patients were from Korea while the rest were from Hong Kong and the Philippines (22.8% each). The study population was predominantly female (75.4%), with a mean age of 57.0 (SD 13.4) years. Common causes of musculoskeletal pain were osteoarthritis (48.3%) and low back pain (37.7%). Sixty-two percent of patients had moderate pain (BS-11 score 4 to 6) and 38.1% had severe pain (BS-11 score ≥ 7) at study entry. The majority of patients (81.6%) reported at least one concomitant illness during the study period. Patients’ demographics and characteristics varied across the countries or territories. The Philippines tended to have a higher mean age (62.2 [SD 11.3]) years and a higher proportion of female patients (84.6%) than Hong Kong and Korea. In Hong Kong, patients reported a fairly even distribution of pain arising from a range of musculoskeletal conditions, with a trend towards higher frequency of low back pain (34.6%). In Korea, low back pain (51.6%) and osteoarthritis (46.8%) were more common while osteoarthritis (76.9%) was the most common cause of chronic pain in the Philippines. Hong Kong had the highest proportion of patients (96.2%) who had concomitant illnesses across countries or territories.

**Table 1:** Patient demographics and characteristics at study entry

Characteristics	Safety population			
	Hong Kong (*n* = 26)	Korea (*n* = 62)	Philippines (*n* = 26)	All (*n* = 114)
Age (years), mean (SD)	55.2 (7.7)	55.6 (15.5)	62.2 (11.3)	57.0 (13.4)
Gender, *n* (%)				
Male	8 (30.8)	16 (25.8)	4 (15.4)	28 (24.6)
Female	18 (69.2)	46 (74.2)	22 (84.6)	86 (75.4)
Causes of pain, *n* (%)				
Osteoarthritis	6 (23.1)	29 (46.8)	20 (76.9)	55 (48.3)
Rheumatoid arthritis	6 (23.1)	0 (0.0)	2 (7.7)	8 (7.0)
Low back pain	9 (34.6)	32 (51.6)	2 (7.7)	43 (37.7)
Joint or muscle pain	5 (19.2)	1 (1.6)	2 (7.7)	8 (7.0)
^a^Concomitant illnesses, *n* (%)				
Yes	25 (96.2)	48 (77.4)	20 (76.9)	93 (81.6)
No	1 (3.8)	14 (22.6)	6 (23.1)	21 (18.4)

*SD* standard deviation

^a^Concomitant illness was defined as any medical condition, other than the primary condition, that occurred within the last five years prior to study entry or during the course of the study

### Exposure

**Fig. 3:**
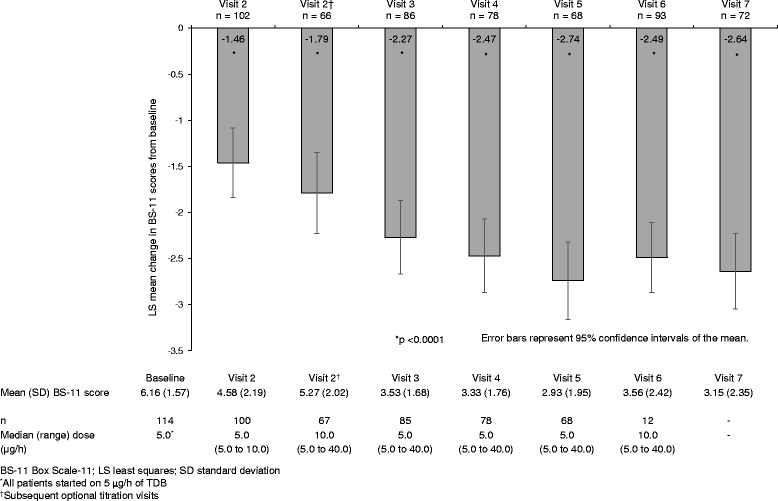
Change in BS-11 scores from baseline to visit 7 (ITT population)

Patients received TDB for a median duration of 12.1 (range 0.3 to 19.1) weeks, at a median dose of 5.0 (5.0 to 40.0) μg/h over the study period. All patients were initiated on TDB at a median dose of 5.0 μg/h. The dose during titration was 5.0 (range 5.0 to 10.0) μg/h at visit 2 (*n* = 100) and was titrated to 10.0 (range 5.0 to 40.0) μg/h among those who required additional titration (*n* = 67). It decreased to 5.0 (range 5.0 to 40.0) μg/h at visit 3 and was maintained at the same dose for the rest of the treatment period (Fig. 3). The most frequently prescribed dose over the study period was 5 μg/h (65.1% of total prescriptions), followed by 10 μg/h (22.7%), and 15 μg/h (7.4%). Other doses were less frequently used: 20 μg/h (2.5%), 25 μg/h (1.0%), 30 μg/h (0.4%), and 40 μg/h (1.0%).

### Efficacy

The results of all efficacy analyses conducted in the ITT population were consistent with those of the PP analyses. Therefore, only results of the ITT population are presented here.

#### Change in BS-11 pain score

Figure 3 summarizes the change in BS-11 scores from baseline to the end of study in the ITT population. The mean BS-11 score at baseline was 6.2 (SD 1.6). Following initiation of TDB treatment, there was a statistically significant improvement in BS-11 score from baseline to visit 3, which was maintained till the end of the study (visit 7) (*p* < 0.0001 for all). The corresponding LS mean change in score from baseline was −2.27 (95% CI -2.66 to −1.87) at visit 3 and −2.64 (95% -3.05 to −2.23) at visit 7.

#### Improvements in sleep quality and quality of life

A marginally significant improvement (*p* = 0.054) in overall sleep quality assessment was observed at visit 6. The proportion of patients rating sleep quality as ‘good’ increased from 14.0% at baseline to 26.9% at visit 6. Patients reported a median sleep duration of 6.0 (range 2.0 to 9.0) hours per night over the seven days prior to the start of treatment. There was no clear improvement in sleep duration from baseline at visit 6. Patients reported minimal or low degree of sleep disturbance for all six variables at baseline (Table 2). By visit 6, the median sleep disturbance scores for the sleep variables “trouble falling asleep” and “awakened by pain in the morning” improved significantly by one unit from baseline (*p* < 0.0001 for both). There were no clear improvements in scores for the other sleep variables at visit 6 (Table 2).

**Table 2:** Improvements in GSQA scores from baseline to visit 6

GSQA Variables		ITT population (*n* = 114)					
		Trouble falling asleep	Need pain medication to sleep	Need sleep medication to sleep	Awakened by pain at night	Awakened by pain in the morning	Effect of pain on partner’s sleep
Baseline	*n*	114	114	114	114	114	86
	Median (range)	3.00 (0.00 to 10.00)	1.00 (0.00 to 10.00)	0.00 (0.00 to 10.00)	2.00 (0.00 to 10.00)	2.00 (0.00 to 10.00	1.00 (0.00 to 10.00)
	Mean (SD)	3.73 (3.18)	2.30 (2.96)	0.91 (2.13)	3.09 (3.19)	2.96 (3.41)	2.71 (3.42)
	Missing	0	0	0	0	0	28
Visit 6	*n*	93	93	93	93	93	80
	Median (range)	1.00 (0.00 to 10.00)	0.00 (0.00 to 10.00)	0.00 (0.00 to 10.00)	1.00 (0.00 to 10.00	2.00 (0.00 to 10.00	0.00 (0.00 to 10.00
	Mean (SD)	2.34 (2.85)	1.74 (2.86)	0.91 (2.13)	2.30 (2.89)	2.96 (3.41)	1.69 (2.60)
	Missing	21	21	21	21	21	34
^a^Change	*n*	93	93	93	93	93	70
	^†^ *p*-value	<0.0001	0.001	0.578	0.004	<0.0001	0.007
	Median (range)	−1.00 (−10.00 to 5.00)	0.00 (−10.00 to 7.00)	0.00 (−10.00 to 10.00)	0.00 (−10.00 to 7.00)	−1.00 (−10.00 to 7.00)	0.00 (−10.00 to 5.00)
	Mean (SD)	−1.68 (3.15)	−0.86 (2.83)	−0.14 (2.85)	−1.04 (3.28)	−1.53 (3.27)	−1.09 (3.08)
	Missing	21	21	21	21	21	44

*GSQA* Global Sleep Quality Assessment Scale, *ITT* intent-to-treat, *SD* standard deviation

^a^Only patients with non-missing data at both visits 1 and 6 are included in the calculation for the change in scores from visit 1

^†^Wilcoxon signed-rank test

**Fig. 4:**
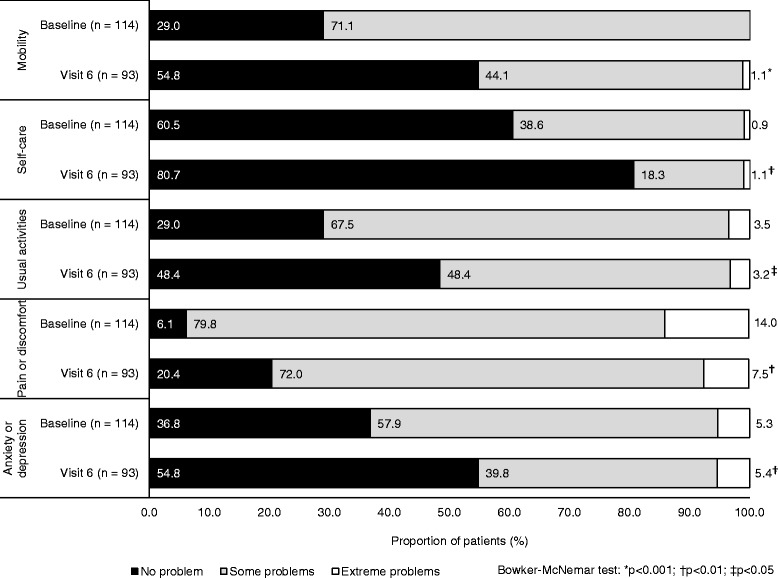
Comparison of patients’ levels of functioning for individual EQ-5D-3 L dimensions between baseline and visit 6 (ITT population)

The mean EQ VAS score at baseline was 59.6 (SD 15.9). Patients’ overall health state improved significantly from baseline (*p* < 0.0001) after treatment. By visit 6, the mean EQ VAS score had increased by 7.7 units (SD 17.9). There were also significant improvements in patients’ levels of functioning for all EQ-5D-3 L dimensions from baseline at visit 6 (*p* < 0.05 for all) (Fig. 4). The proportion of patients who indicated they had no problem with each dimension increased from baseline to visit 6: mobility (from 29.0% to 54.8%), self-care (from 60.5% to 80.7%), usual activities (from 29.0% to 48.4%), pain or discomfort (from 6.1% to 20.4%), anxiety or depression (from 36.8% to 54.8%) (Fig. 4).

#### Patient and physician global assessment of pain relief

There was no significant difference between patients’ and physicians’ Global Impression of Change scores at visit 6 (*p* = 0.248). The median Global Impression of Change score was 3.0 (range 1.0 to 5.0) for both patients and physicians, indicating perceived improvement in overall pain condition at the end of TDB treatment.

#### Rescue medication use

Only 22.8% of patients required rescue medications during the study period. The most frequently prescribed rescue medications were acetaminophen (56.1% of total prescriptions), followed by diclofenac (23.6%). Other medications were less frequently used (0.8–7.3%).

### Safety

Overall, eighty-nine patients (78.1%) reported TEAEs, most of which were mild to moderate in intensity (96.5%). The most common TEAEs reported during the study were nausea (39.5%) and constipation (31.6%), followed by dizziness (27.2%), somnolence (19.3%), vomiting (16.7%), headache (8.8%), pruritus (7.9%), and -application site reactions (6.1%). Sixty-eight percent of patients had TEAEs that were assessed to be related to the study medication by the physician. One serious TEAE was reported during the course of the study. A patient experienced a hypertensive crisis; the event was moderate in severity but was not considered related to the study medication. The incidence of TEAEs leading to discontinuation was 22.8%, most of the events were mild-moderate in intensity (92.9%). TEAEs that frequently led to discontinuation of the study medication included nausea (11.4%), dizziness (7.9%), and vomiting (5.3%).

Table 3 summarizes the incidence of TEAEs and common TEAEs, as well as the practice pattern in AE management of TDB treatment in the participating countries or territories. The incidence of TEAEs varied across countries or territories: Korea had a lower incidence of TEAEs (69.4%) than the Philippines (80.8%) and Hong Kong (96.2%). A similar trend was also observed for discontinuation due to TEAEs, with a lower incidence in Korea (14.5%) than the Philippines (26.9%) and Hong Kong (38.5%). Physicians more frequently prescribed medications to treat AEs that occurred during TDB treatment than to manage them prophylactically (Table 3). Only 14.9% of patients received prophylactic medications while 23.7% received medications for treatment of AEs. Antiemetics were the most common preemptive treatment prescribed while both antiemetics and laxatives were commonly prescribed to treat AEs. Of note, physicians in Korea, the country with the lowest incidence of TEAEs, tended to prescribe medications both to prevent (24.2%) and treat (22.6%) AEs. In contrast, physicians in the Philippines and Hong Kong tended to prescribe medications to treat AEs (19.2% and 30.8%, respectively) rather than to prevent their occurrence (0.0% and 7.7%, respectively) (Table 3).

**Table 3:** Incidence of TEAEs and common TEAEs, and AE management of TDB treatment

	Safety population			
	Hong Kong (*n* = 26) *n* (%)	Korea (*n* = 62) *n* (%)	Philippines (*n* = 26) *n* (%)	All (*n* = 114) *n* (%)
Incidence of TEAEs	25 (96.2)	43 (69.4)	21 (80.8)	89 (78.1)
TEAEs leading to discontinuation	10 (38.5)	9 (14.5)	7 (26.9)	26 (22.8)
^a^Common TEAEs				
Nausea	11 (42.3)	27 (43.6)	7 (26.9)	45 (39.5)
Constipation	10 (38.5)	20 (32.3)	6 (23.1)	36 (31.6)
Dizziness	13 (50.0)	9 (14.5)	9 (34.6)	31 (27.2)
Somnolence	9 (34.6)	8 (12.9)	5 (19.2)	22 (19.3)
Vomiting	9 (34.6)	4 (6.5)	6 (23.1)	19 (16.7)
Headache	4 (15.4)	2 (3.2)	4 (15.4)	10 (8.8)
Pruritus	4 (15.4)	4 (6.5)	1 (3.9)	9 (7.9)
Application site reactions	3 (11.5)	0 (0.0)	4 (15.4)	7 (6.1)
^b^AE management of TDB treatment				
Received medications for prevention and/or treatment of common AEs	10 (38.5)	26 (41.9)	5 (19.2)	41 (36.0)
^c^Prevention	2 (7.7)	15 (24.2)	0 (0.0)	17 (14.9)
Antiemetics	1 (3.9)	15 (24.2)	0 (0.0)	16 (14.0)
Laxatives	1 (3.9)	0 (0.0)	0 (0.0)	1 (0.88)
^d^Treatment	8 (30.8)	14 (22.6)	5 (19.2)	27 (23.7)
Antiemetics	2 (7.7)	11 (17.7)	2 (7.7)	15 (13.2)
Laxatives	5 (19.2)	3 (4.8)	5 (19.2)	13 (11.4)
Antivertigo agents	3 (11.5)	0 (0.0)	0 (0.0)	3 (2.6)

*AE* adverse event, *TDB* transdermal buprenorphine, *TEAE* treatment-emergent adverse events

^a^Occurring in ≥5% of the overall safety population

^b^Patients may receive one or more medications for AE management

^c^Prescribed on the same date as the start date of TDB treatment

^d^Prescribed after the start of TDB treatment

There were no clinically significant changes in vital signs or physical examination results between baseline and the end of the study.

## Discussion

This is the first multinational study of TDB treatment in Asian patients who were suffering from moderate to severe pain due to a range of musculoskeletal conditions. This study, reflecting real-world clinical practice, demonstrates that TDB provides effective pain relief and improves daily functioning and quality of life in Asian patients with chronic musculoskeletal pain after 11 weeks of treatment. Additionally, TDB demonstrates an acceptable tolerability profile in our Asian cohort over the treatment period, consistent with that observed in Caucasians.

In the present study, patients entered the study with an average BS-11 pain score of 6.2. After initiation of TDB, the mean BS-11 score decreased by 2.3 unit one week after the end of the titration period and the reduction was maintained till the end of the treatment period (LS mean change: −2.5 at visit 6). A reduction of approximately 2 units from baseline on the 11-point numeric rating scale has been demonstrated to correspond to a clinically meaningful improvement [31]. The improvements in BS-11 score observed in this study are therefore considered clinically relevant. In addition, the majority of patients did not require additional rescue medications for pain. Similar improvements were observed in an open-label, randomized trial conducted in UK [23]. In this trial, reduction in BS-11 score was sustained over the 11-week treatment period (mean reduction: 3.6 at end of titration and 4.0 at week 12) in patients with knee and/or hip osteoarthritis who received TDB plus oral paracetamol [23]. The benefits of TDB in providing analgesia in patients with chronic moderate to severe non-malignant pain are supported by results from other studies [25, 26]. In a prospective study of younger patients and elderly patients with osteoarthritis-related pain in Sweden, Karlsson et al. observed significant reductions in BS-11 score in patients who were treated with TDB, regardless of age (LS mean change: −1.9 to −2.2). In addition, patients used less rescue medications after the start of the 12-week treatment. The mean number of tablets of rescue medication taken each day reduced from 5.2 to 5.7 at baseline to 2.1 to 2.8 during the treatment period [26]. In another open-label, randomized study comparing the efficacy and safety of TDB with prolonged-release tramadol tablets, similar improvement in BS-11 score (LS mean change: −2.3) was reported in Swedish patients after 12 weeks of treatment with TDB [25].

Significant differences in the metabolism and response to medicines have been documented among racial groups [32]. The prescribed TDB dose was lower in this study compared to other studies conducted in Caucasian patients [24, 26, 33]. The median dose for the last dose prescribed during the treatment period in this study was 5 μg/h while higher doses (10–24 μg/h) were prescribed in other studies [24, 26, 33]. This findings suggest that Asian patients may require lower doses of TDB than Caucasian patients. More studies are needed to establish the dose level of TDB for treatment of pain in Asian patients.

The goal of chronic pain management encompasses not only the relief of pain, but also maintaining or improving the daily functioning and quality of life of patients [2]. In the present study, in addition to reduction of pain intensity, patients’ quality of life and levels of functioning improved at the end of treatment with TDB. The proportion of patients who rated sleep quality as ‘good’ on the 8-item GSQA scale increased after treatment. Patients reported improvement in overall health state (EQ VAS) after treatment. Improvements in patients’ levels of functioning for all dimensions of the EQ-5D-3 L questionnaire were also observed. Similar results were observed in previous studies of TDB [25, 26]. In the study by Karlsson et al., patients reported fewer nights of sleep disturbance. The EQ VAS score increased by an average of 6.8 at study completion [26]. In another study, the majority of patients (71%) reported improved ratings in sleep quality at the end of treatment. Improvement in EQ VAS score at study completion was also observed [25].

Consistent with the decrease in pain scores in the present study, both physicians and patients rated TDB treatment as associated with improvement in the overall pain condition. Previous studies of TDB have also reported favorable physician and patient ratings for TDB treatment [25, 26]. In the recent study by Karlsson et al., the majority of physicians (61 to 65%) and patients (59 to 68%) rated TDB as “good” or “very good” at relieving pain [26]. Similar high proportions were reported in another study (physicians: 72.1%, patients: 64.7%, respectively) [25].

No unexpected safety or tolerability concerns were raised in the present study. Although the incidence of AEs and the incidence of discontinuations due to AEs were high, these were not unexpected as the incidence rates were consistent with those reported in previous studies of TDB conducted in Caucasian patients (incidence of AEs: 86 to 91% and incidence of discontinuations: 15 to 35%) [23–26]. As with previous studies, AEs were generally mild or moderate. The most common AEs reported were consistent with the AE profile of TDB in general [23–26]. Of note, the incidence of skin reactions reported in this study was lower than that reported in other studies (27 to 30%) [23, 24]. As with previous studies [23, 25, 26], no significant abnormalities in vital signs or physical examination parameters were reported during the study.

Clinical guidelines from the American Pain Society-American Academy of Pain Medicine and the European Association for Palliative Care (EAPC) recommend physicians to employ preemptive intervention or symptomatic management of AEs to minimize the common side effects of opioid therapy [34, 35]. In the present study, prescription of medications for prevention or treatment of common AEs of TDB treatment was uncommon despite the high incidence of AEs reported. Although guidelines recommend to routinely prescribe laxative for prophylaxis management of opioid-induced constipation [34, 35], prescription of prophylactic laxative treatment was low in this study. These findings indicate a significant gap between guideline recommendations and the actual practice in managing the side effects of opioid treatment in the participating countries or territories. The development of education and training strategies to improve physicians’ knowledge and to raise their awareness on opioid treatment may lead to improvements in treatment adherence.

In the present study, the incidence of AEs and the incidence of discontinuations varied across the countries or territories. The incidence rates appeared consistent with the practice pattern in side effect management of TDB treatment in each country or territory. Lower incidence rates were observed in Korea where medications for management of common AEs were prescribed both prophylactically and after TDB treatment. In contrast, higher incidence rates were observed in Hong Kong and the Philippines where physicians tended to prescribe medications to treat AE rather than to prevent their occurrence. These findings highlight the importance of preemptive and active management of side effects of TDB treatment in supporting treatment adherence. While practice pattern in side effect management influence the incidence rates, it is possible that ethnic differences in the development of AEs may exist. More studies are needed to confirm if the varying AE profile across the countries or territories is due to ethnic differences. This will provide valuable information for physicians to properly manage the use of TDB for pain management in different ethnic groups.

Our findings need to be interpreted within the limitations of the study. First, the treatment duration of 11 weeks is relatively short, and whether the observed treatment effect continues in the longer term would require further research to confirm. Next, the GSQA questionnaire has not been formally validated. Nevertheless the use of a structured questionnaire to assess sleep disturbance and sleep quality ensured consistency in data collection before and after TDB treatment and minimized bias to some extent. Another limitation is that the study population comprised a heterogeneous mix of musculoskeletal conditions with distinct pathophysiological mechanisms. It is probable that some conditions responded better than others to the treatment. However, the sample size is too small to allow parsing of the results by musculoskeletal condition. Future studies in additional populations of patients are needed to evaluate the effect of TDB in different conditions. In addition, there is potential for bias due to the open-label study design and the lack of active control in this study which preclude evaluation of the extent to which the observed effect is caused by the treatment. Nonetheless, the present study closely reflects the real-life clinical setting and the results are more likely to translate effectively into real-world clinical practice.

## Conclusions

Our study provides real-world clinical evidence on the effectiveness and tolerability of TDB in Asian patients with moderate to severe chronic pain. Treatment with TDB resulted in effective and sustained pain relief over the 11-week treatment period, accompanied by improvements in daily functioning and quality of life. The tolerability profile was as expected as previous studies of TDB. Our results indicate that TDB can be considered a suitable alternative treatment option to control non-malignant musculoskeletal pain. Studies on the long-term efficacy and safety of TDB treatment are required to further confirm these findings.

## Abbreviations

AEs: Adverse events
BS-11: Box Scale-11
CIs: Confidence intervals
EAPC: European Association for Palliative Care
EQ VAS: EQ-5D visual analogue scale
EQ-5D-3 L questionnaire: EuroQol Group 5-Dimension Self-Report Questionnaire-3 Level Version Survey
GSQA: Global Sleep Quality Assessment Scale
ITT: Intent-to-treat
LS: Least squares
NICE: National Institute for Health and Care Excellence
NSAIDs: Nonsteroidal anti-inflammatory drugs
PP: Per-protocol
SD: Standard deviation
TDB: Transdermal buprenorphine
TEAEs: Treatment-emergent adverse events
